# Establishment and Functional Characterization of Murine Monoclonal Antibodies Recognizing Neuritin

**DOI:** 10.3390/antib12020028

**Published:** 2023-04-07

**Authors:** Georgia Papadogianni, Inga Ravens, Ahmed Hassan, Andrew Flatley, Regina Feederle, Günter Bernhardt, Hristo Georgiev

**Affiliations:** 1 Institute of Immunology, Hannover Medical School, 30625 Hannover, Germany; 2 Monoclonal Antibody Core Facility, Helmholtz Center Munich, Research Center for Environmental Health (GmbH), 85764 Neuherberg, Germany

**Keywords:** neuritin, blocking antibody, flow cytometry, ELISA, monoclonal antibody

## Abstract

Neuritin represents a neurotrophic factor that is not only important in neuronal development and plasticity but also impacts endothelial angiogenesis, cell migration, tumor growth and the production of antibodies by B cells. We established monoclonal mouse anti-mouse neuritin antibodies by immunizing knock-out mice with two different neuritin-derived peptides. Because neuritin is well conserved between species, these new monoclonal antibodies recognize the neuritin of a wide variety of species, including human. Moreover, they not only recognize specifically surface-bound neuritin expressed by murine follicular regulatory T cells but also the block binding of recombinant neuritin to germinal center B cells. This suggests that these newly generated tools will be of great use in studying neuritin expression and function.

## 1. Introduction

Neuritin was discovered in the rat as a neuropeptide that is involved in neuronal plasticity [1,2,3]. It is expressed either as a GPI-anchored membrane protein or is released by cells, most likely by phospholipase C-triggered shedding [4]. Neuritin promoted cell growth, neurite outgrowth and arborization [1,2,3]. Moreover, in several studies it counteracted apoptosis, e.g., in cortical progenitor cells [4] or in the visual cortex [5], respectively. In addition, neuritin assisted in repairs following nerve injury [6,7]. The absence of neuritin provoked several neuronal phenotypes [5,8]; the role of this neuropeptide in Alzheimer’s disease, as well as in psychiatric disorders, is discussed [9].

The expression of neuritin is not restricted to the nervous system. Noticeable amounts of mRNA-coding human neuritin was observed in several tissues such as placenta, lung, thymus, liver and others [10]. Indeed, recent research revealed additional functions of neuritin, e.g., it promoted the migration of endothelial cells and angiogenesis [11]. Along this line, neuritin overexpression increased the migration of bone marrow mesenchymal stem cells via a CXCR4-driven mechanism [12]. Interestingly, the expression of neuritin is coupled positively with that of CXCR4. In clear cell renal cell carcinoma, the forced downregulation of neuritin expression in patient-derived spheroid cultures decreased CXCR4 levels and suppressed tumor cell growth [13]. Thus, neuritin is also involved in tumor biology and several other studies have confirmed such a role [10,13,14,15,16]. Most recently, evidence was provided that neuritin, produced by follicular regulatory T (TFR) cells, impacts differentiating B cells, thereby manipulating their antibody production [17]. Neuritin suppressed the ample production of auto-reactive antibodies and influenced an isotype switch to antibody isotypes IgE and IgG1, thereby assisting in the prevention of autoimmunity and allergy [17].

The growing spectrum of diverse neuritin functions makes this neurotrophic factor an increasingly attractive subject of research in several fields. In medicine it can serve as a diagnostic tool and may represent a target for therapeutic interventions. Such perspectives necessitate the need for suitable tools to detect neuritin expression and to manipulate its functions. We report here the establishment of mouse anti-neuritin monoclonal antibodies (mAb) generated by the use of neuritin-deficient hosts. The mAbs specifically detect mouse and human neuritin by flow cytometry assay and possess a neutralizing capacity.

## 2. Materials and Methods

### 2.1. Mice

The neuritin-deficient mouse strain B6, FVB-Nrntm1.2Ndiv (stock No 018402), was purchased from Jackson Laboratory. A breeder colony was established at the animal facility of Medizinische Hochschule Hannover (MHH). Monitoring of the ko-status was done as suggested by the supplier. The B6 animals were obtained from in house breeding. Animal experiments were conducted according to MHH guidelines and approved by LAVES, Lower Saxony, as well as the government of Upper Bavaria (ROB-55.2-2532.Vet_03-17-68).

### 2.2. Cloning, Expression and Purification of Recombinant Protein

The full-length coding sequences (CDS) of murine (NM_153529.2) and human (AF136631.1) neuritin (NRN1) were ordered as gBlocks (Integrated DNA Technologies) and subsequently cloned into lentiviral expression vector lenti-EGFP [18]. In addition, the following construct was generated for the expression and purification of recombinant NRN1: murine NRN1 CDS fused via a GAG linker to the FLAG tag (DYKDDDDK) followed by Twin-Strep-tag [19]. The generated construct was further subcloned into the lentiviral expression vector (lenti-mNeuritin-FLAG). The lenti-mNeuritin-FLAG construct was used to transfect HEK293 cells (see following section) and the supernatant was collected 2 days later. The recombinant protein was purified from the supernatant by Strep-Tactin^®^XT 4Flow^®^ columns (IBA-Lifesciences, Göttingen, Germany), following the protocol provided by the manufacturer.

### 2.3. Transient Transfection of HEK293 Cells

HEK293 cells were grown in DMEM/10%FBS and transfected, applying the standard calcium phosphate procedure. Two days later, cells were harvested and analyzed. Alternatively, culture supernatants were collected and recombinant proteins were purified.

### 2.4. Establishment of Monoclonal Antibodies

Two peptides comprising either amino acids 47DSMANYPQGLDDKTN61 (NRNA) or 83CQEGAKDMWDKLRKESKN100 (NRNB) of mouse neuritin1 protein were synthesized and coupled via N-terminal cysteine with ovalbumin (Ova) and biotin (Peps4LS, Heidelberg, Germany). Nrn1 knock-out mice were immunized with a mixture of 40 µg Ova-coupled peptides, 6 nmol CpG oligonucleotides (Tib Molbiol, Berlin, Germany) in 200 µL PBS and 200 µL incomplete Freund’s adjuvant. A boost without adjuvant was given 14 weeks after the primary injection. The fusion of mouse spleen cells with myeloma cell line P3 × 63-Ag8.653 (ATCC, American Type Culture Collection) was performed using standard procedures. Hybridoma supernatants were tested in a flow cytometry assay (iQue, Intellicyt; Sartorius, Gottingen, Germany) for binding to biotinylated neuritin1 peptides coupled with streptavidin beads (PolyAN, Berlin, Germany). Using Atto-488-coupled isotype-specific monoclonal rat-anti-mouse IgG secondary antibodies (anti-IgG3 (clone 2E.6, HB-128, ATCC), all other in house), antibody binding was analyzed using ForeCyt software (Sartorius). Positive supernatants were further analyzed by flow cytometry analysis and selected hybridoma cells were subcloned twice by limiting dilution to obtain stable monoclonal cell lines. Experiments in this work were performed with the hybridoma supernatant of clones NRNA 30A12 (mouse IgG2b), NRNA 32D12 (mouse IgG2a) and NRNB 27G7 (mouse IgG2a). mAbs were purified using a Protein A Sepharose Fast Flow resin (Cytiva, Marlborough, MA, USA) and subsequent gel filtration (Superdex 200 pg; Cytiva). Purified mAbs were stored at 4 °C in PBS buffer. Purified mAbs were biotinylated (EZ-Link NHS-LC-Biotin; ThermoFisher Scientific, Waltham, MA, USA) or conjugated with horseradish peroxidase (HRP conjugation kit; Abcam, Cambridge, UK).

### 2.5. Immunization of Mice

For immunization, a cocktail was prepared consisting of 200 µg 4- Hydroxy-3-nitrophenylacetyl hapten bound to Keyhole Limpet Hemocyanin (NP-KLH, BioCat, Barcelona, Spain) and 50 µL 2% alum (InvovoGen, San Diego, CA, USA) in a total volume of 100 µL. Twelve days later, the draining lymph nodes were harvested and cell suspensions were prepared.

### 2.6. Production of Fab Fragments

Fab fragments of mAb 27G7 and 32D12 were obtained using immobilized papain (ThermoFisher Scientific), according to the manufacturer’s protocol, and further purified by removing Fc parts and undigested mAb by overnight incubation with MabSelect PrismA beads (Cytiva).

### 2.7. Enzyme-Linked Immunosorbent Assays

Recombinant human/rat neuritin (Peprotech, Cranbury, NJ, USA) was bound to 96-well plates (5 or 10 ng per well in PBS in duplicate) overnight at 4 °C and washed three times. All following incubation steps were performed for 50 min at 37 °C. Blocking was done with 3% non-fat dry milk in TBST and the plates washed with TBST before serial dilutions of mAbs (either purified or HRP-labeled) in TBST were added. Purified mAbs were detected by an additional incubation with HRP-labeled isotype-specific rat anti-mouse IgG2a or IgG2b mAbs (in house), before all plates were washed six times with TBST prior to the addition of a TMB substrate solution (BD Biosciences, East Rutherford, NJ, USA). Color development was stopped by adding 0.5 N sulfuric acid.

### 2.8. Flow Cytometry

Single-cell suspensions were prepared and blocked with TruStain FcX™ PLUS Antibody (BioLegend, San Diego, CA, USA). All surface stainings were performed for 30 min on ice. Intracellular staining for FOXP3 was conducted using a FOXP3 staining buffer (eBioscience, San Diego, CA, USA), according to the protocol provided by the manufacturer. The antibodies used are as follows: anti-mCD19 (clone 6D5), anti-mGL-7 (clone GL-7), anti-FLAG-Tag (clone L5) and streptavidin (APC) were from BioLegend; anti-mPD-1 (clone J43), anti-mFoxp3 (clone FJK-16S) and anti-mCD95(Fas) (clone 15A7) were from eBioscience; anti-mCD4 (clone RM4-5) was from (BD Bioscience). Surface stainings with purified 27G7, 30A12 and 32D12 mAbs were detected with AffiniPure F(ab’)_2_ fragment donkey anti-mouse IgG (H+L) from Jackson ImmunoResearch Laboratories. Data were acquired on LSR II (Becton Dickinson) or on an Aurora spectral flow cytometer (Cytek, San Diego, CA, USA) and analyzed with FlowJo version 10.1r5 (Tree Star, Ashland, OR, USA).

### 2.9. Statistical Analysis

All statistical analyses were performed with GraphPad Prism 8.

## 3. Results

### 3.1. Establishment and Validation of Mouse Anti-Neuritin Monoclonal Antibodies (mAbs)

Upon comparison of the transcriptomes of murine T cell subsets present in secondary lymphoid organs, we noted that the mRNA coding for neuritin was upregulated in follicular T cells [20]. In particular, a strong induction of expression was observed in TFR cells in the draining lymph nodes of immunized animals. However, it was not possible to corroborate this interesting finding by flow cytometry because suitable antibodies were not available. Therefore, we intended to establish monoclonal anti-neuritin antibodies. A routine procedure using rats as hosts to obtain rat anti-mouse monoclonals appeared futile, since neuritin is very well conserved among species (Figure S1). Indeed, initial efforts to study the function of neuritin included Xenopus laevis neurons that were treated with neuritin of rat origin [3]. Therefore, we used neuritin-deficient mice as hosts to establish monoclonal anti-neuritin antibodies. Protein sequence analysis was performed on the Husar (Heidelberg Unix Sequence Analysis Resources) server (DKFZ Heidelberg) using the method for calculating antigenic index as described by Jameson and Wolf [21]. Two peptides, NRNA (DSMANYPQGLDDKTN) and NRNB (CQEGAKDMWDKLRKESKN), were selected based on the predicted antigenicity. Primary hybridoma culture supernatants were first screened for binding to bead-coupled biotinylated peptides in a flow cytometry assay and positive supernatants were further validated for binding to transiently transfected HEK293 cells expressing murine neuritin by FACS and also to recombinant neuritin in ELISA (data not shown). Based on these data, three hybridomas were selected for subcloning by limiting dilution to establish monoclonal cell lines: clones 30A12 (IgG2b) and 32D12 (IgG2a) raised against peptide NRNA and clone 27G7 (IgG2a) specific for peptide NRNB. A criterion for selecting the two clones 30A12 and 32D12 as candidates recognizing peptide NRNA was due to their diverging isotype.

**Figure 1:**
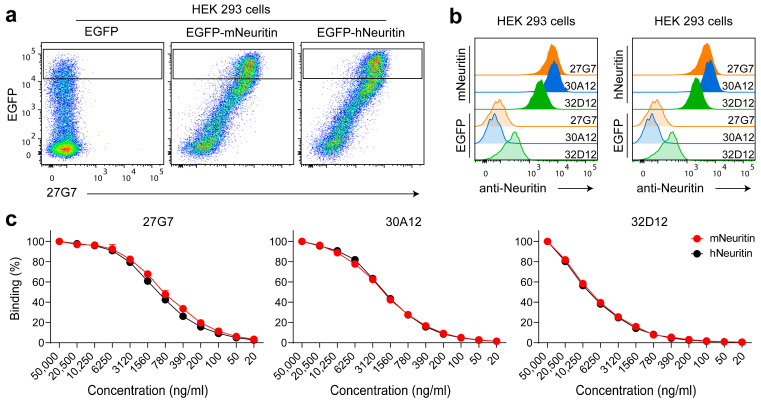
Detecting binding of purified mAb to transiently expressed neuritin by flow cytometry. (**a**) Shown is the binding of clone 27G7 to a suspension of 100 µL HEK293 cells transiently expressing neuritin and EGFP or to cells expressing EGFP only. (**b**) Comparison of fluorescence intensities provoked by binding of one µg mAb 27G7, 30A12 and 32D12, respectively, to cells shown in the gates in (**a**). (**c**) Titration of the mAb clones on HEK293 cells expressing either human or murine neuritin; gates were set as shown in (**a**). Highest fluorescence intensities at concentrations or 5 µg mAb per 100 µL of sample were set to 100 and all other intensities calculated as percentages of these values. Representative plots are shown in (**a**,**b**). (**c**) Represents the summary of two independent titration assays. Shown are means ± SD.

We investigated the functionality of the subcloned and purified mAbs in subsequent experiments. The purified mAbs bound to HEK293 cells overexpressing murine neuritin; however, signal intensity and background staining varied (Figure 1a,b). mAb 30A12 yielded the highest signal intensity, but mAb 27G7 displayed the highest avidity (see titration in Figure 1c). As expected, the three mAbs bound both human and mouse neuritin equally well because the amino acid composition of both peptides used to generate them are identical in the two species (Figure 1 and Figure S1). Since this is the case for a wide variety of species (Figure S1), a broad species reactivity can be expected.

### 3.2. Application of the mAbs in ELISA Assays

**Figure 2:**
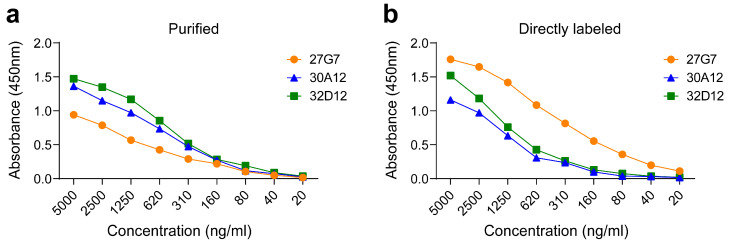
Binding of mAb to plate-bound recombinant neuritin in ELISA. (**a**) ELISA was performed with serial dilutions of purified mAbs binding to 10 ng of plate-bound neuritin per well. Bound mAb was revealed by HRP-labeled secondary antibody. (**b**) ELISA was performed with serial dilutions of directly HRP-labeled mAb binding to 5 ng neuritin per well. Shown are representative ELISA tests of at least two independent experiments each.

Next, we analyzed the binding capabilities of the mAbs to recombinant neuritin protein by ELISA (Figure 2). When binding was detected using a HRP-labeled secondary conjugate (Figure 2a), clone 32D12, and also 30A12, displayed a stronger binding profile compared with clone 27G7, most probably reflecting the diverging microenvironments of plate-bound versus cell-surface-expressed neuritin. For practical issues, it is of interest to use directly labeled mAbs in experiments; therefore, we conjugated the mAbs with either HRP or biotin. In ELISA, we observed that mAb 27G7 performed substantially better compared with mAb 30A12 or 32D12 (Figure 2b). It appeared that both clones specific for peptide A, but not for clone 27G7 recognizing peptide B, lost some antigen affinity upon covalent conjugation with HRP. A reduction or even loss of binding capability is a frequently met problem when antibodies are modified covalently. Since conjugation was performed via non-specific coupling of HRP with lysine side chains, the reduced binding capability is most likely due to modified lysines in or close to the antibody binding sites.

### 3.3. Clones 27G7, 30A12 and 32D12 Recognize Surface-Bound Neuritin Expressed by Murine Follicular Regulatory T

**Figure 3:**
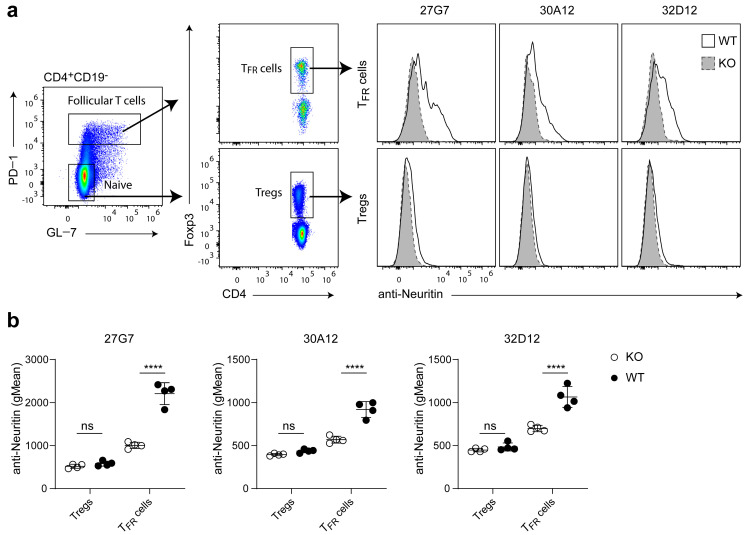
Ex vivo analysis of T cell subsets present in draining lymph nodes of immunized mice. CD4+CD19- T cells from draining lymph nodes 12 days post immunization were analyzed for expression of surface-bound neuritin. (**a**) Left and middle panels show the gating strategy allowing analysis of Treg cells and TFR cells. Histograms to the right show the binding of mAb clones 27G7, 30A12 and 32D12 to TFR cells (upper row) and Treg cells (lower row). Wild-type cells: solid lines; cells of neuritin-deficient origin: dashed lines. Shown are representative staining profiles. (**b**) Summarizes the data from two independent experiments analyzing TFR cells and Treg cells obtained from a total of four wild-type and four neuritin-deficient mice. gMean—geometric mean. Bars denote mean ± SD (one-way ANOVA followed by Tukey’s multiple comparisons test). n.s.—not significant, **** *p* < 0.0001.

We next used biotinylated mAbs to test whether they can recognize neuritin expressed in the natural context of a cell surface. Previous observations indicated particular upregulation of mRNA-encoding neuritin on TFR cells arising in draining lymph nodes upon immunization of mice. In contrast, neuritin was virtually absent at the mRNA level on naïve CD4 T cells and was only marginally expressed on regulatory T (Treg) cells [20]. To test whether the antibodies can recognize neuritin in the natural context of a T cell surface, we isolated lymph nodes from wild-type C57BL/6 and neuritin knock-out mice 12 days after immunization. In line with the results described above, mAb 27G7 caused a more prominent shift than mAb 30A12 and mAb 32D12 when the neuritin expression of TFR cells was investigated (Figure 3). Regarding Treg cells, only mAb 27G7 caused a marginal shift on wild-type cells when compared with neuritin-deficient Treg cells (Figure 3a). However, this binding did not reach statistical significance (Figure 3b). The results obtained with mAb 27G7 confirm the mRNA data of our earlier study [20] but also indicate that a subpopulation of TFR cells lacked detectable amounts of neuritin on the cell surface (Figure 3a). This may relate to the activation status of TFR cells or reflect a distinct maturation stage. Future research must address this issue.

### 3.4. The mAb Block Binding of Recombinant Neuritin to Germinal Center B Cells

**Figure 4:**
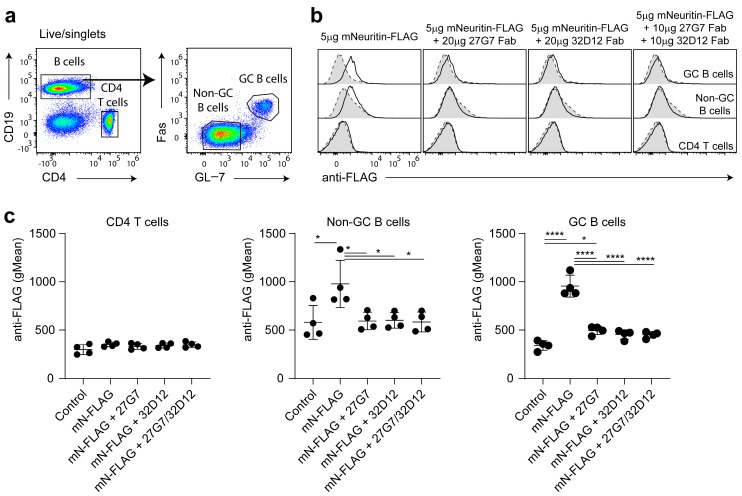
Neutralizing capacity of mAb clones 27G7 and 32D12. Lymphocytes of the draining lymph nodes of immunized B6 mice were gated as shown in (**a**) to define GC B cells, non GC B cells and CD4 T cells, respectively. (**b**) Binding of recombinant FLAG-tagged neuritin to the corresponding cell subsets (left panels) or of the recombinant FLAG-tagged neuritin mixed with the indicated amounts of Fab fragments 30 min prior to addition to the cells. Bound neuritin was detected with an anti-FLAG antibody (solid lines); identical cell samples missing recombinant neuritin served as a negative control (dashed lines). Shown is a representative set of stains, whereas (**c**) summarizes the results from two independent experiments displaying the binding data of recombinant FLAG-tagged neuritin to the indicated cell types obtained from a total of four immunized wild-type B6 mice. Bars denote mean ± SD (one-way ANOVA followed by Tukey’s multiple comparisons test). * *p* < 0.05, **** *p* < 0.0001.

Neuritin affects the humoral immune response (see introduction) via binding to B cells [17]. In the course of an adaptive immune response, GC B cells differentiate from antigen responsive naïve B cells that readily bind and take up neuritin [17]. To test whether our mAbs can interfere with the binding of neuritin to B cells, we produced a recombinant mouse neuritin protein modified at its C-terminus with peptides that allow purification and subsequent detection of neuritin with an anti-FLAG antibody (see experimental procedures). This recombinant protein bound to GC B cells (Figure 4), and to a lesser extent to non-GC B cells, confirms earlier observations [17]. CD4 T cells did not show any neuritin binding (Figure 4). Subsequent initial results of experiments designed to investigate blocking activity of our mAbs were inconclusive due to the overall high background binding to B cells. Therefore, to avoid unspecific binding to Fc receptors abundantly present on B cells, we generated Fab fragments of mAb 27G7 and 32D12. Both Fab fragments were capable of almost completely suppressing the binding of recombinant neuritin to GC B cells and also non-GC B cells (Figure 4b,c).

## 4. Discussion

Although anti-neuritin antibodies are available that work reportedly well in immunohistochemistry (Western blot or ELISA) [22], the antibodies that we generated and characterized in depth in this study offer new qualities. They detect neuritin in ELISA but more importantly by flow cytometry; moreover, they possess neutralizing activity. As expected, the mAbs recognized neuritin of human and mouse origin equally well and, based on sequence identities, we predict that they will be capable of binding specifically to neuritin of various other species. The purified mAb 32D12 appeared most sensitive in ELISA, whereas purified mAb 27G7 was superior in flow cytometry. The latter mAb turned out to be most useful in ELISA and in flow cytometry upon conjugation with either HRP or biotin, respectively. An unrestricted sensitivity and specificity of a mAb upon covalent conjugation with enzymes or fluorochromes represents a major prerequisite for broad use in research. In line with this, we showed that the biotinylated mAbs were capable of detecting neuritin in a natural cellular context, i.e., on the surface of murine TFR cells. To our knowledge, the detection of neuritin by flow cytometry on ex vivo primary cells has not been reported so far. Although the mAb strongly recognized surface-bound neuritin on transiently transfected HEK293 cells expressing either murine or human neuritin, the obtained shift in signal intensity was rather moderate on primary cells. This may be explained by the observation that neuritin can be released from the cell surface by shedding. Indeed, TFR cells were identified as a potent source of neuritin [17,20]. Gonzales-Figueroa et al. demonstrated that B cells, in particular GC B cells, bind and take up soluble neuritin, supporting a role of secreted neuritin in manipulating these cells [17]. Therefore, it is tempting to speculate that TFR cells residing in the GC are induced to shed neuritin, giving rise to a subpopulation of cells that has recently lost surface-bound protein (see Figure 3a).

Interestingly, evidence was provided that both forms of neuritin, surface-bound and secreted neuritin, can serve different functions [9]. For example, membrane-bound neuritin was required to promote neurite arborization [3], while secreted neuritin prevented neuronal apoptosis [4]. Taken together, these findings show that the newly developed anti-neuritin mAbs will be useful in further characterizing cell types that express neuritin on their surface and may support functional studies as they are able to neutralize the binding or neuritin to target cells. This could also be relevant for future therapeutic approaches to treat cancer. Although there is evidence that neuritin expression is downregulated in certain tumor types [10], it has been demonstrated that, in other tumor types, elevated neuritin levels correlate with tumor malignancy [13,14,15,16]. For example, neuritin could induce expression of the chemokine receptor CXCR4 in renal cancer, thereby increasing cell viability and the migratory, i.e., metastatic, potential of the tumor cells [13].

## Data Availability

The data are avaliable from the corresponding author.

## Supplementary Materials

The following supporting information can be downloaded at: https://www.mdpi.com/article/10.3390/antib12020028/s1, Figure S1: Alignment of neuritin amino acid sequences of diverse species.
