# Supplementary material for: Establishment and Functional Characterization of Murine Monoclonal Antibodies Recognizing Neuritin
Source: Antibodies (Basel). 2023 Apr 7;12(2):28. doi: 10.3390/antib12020028 (PMC10123642; doi:10.3390/antib12020028)
Supplement: Supplementary file 1 [file antibodies-12-00028-s001.zip › antibodies-2189924-supplementary.pdf]

Supplementary material

```
                             10        20         30        40        50        60
                             .         .          .         .         .         .
Xenopus             MGLKLSGRYIFLVLAVHLAYLLQAVKA TGKCDAVFKGLSDCMLTLGDKVANYPQDLEEKK
Gallus              MGLKLNGRYISLILAVQIAHLVQAVRA AGRCDAVFRGFSDCLLRLGDNMANYPQDLDDKR

Oryctolagus         MGLKLNGRYISLILAVQIAYLVQAVRA AGKCDAVFKGFSDCLLKLGDSMANYPQGLDDKT
Mus                 MGLKLNGRYISLILAVQIAYLVQAVRA AGKCDAVFKGFSDCLLKLGDSMANYPQGLDDKT
Homo                MGLKLNGRYISLILAVQIAYLVQAVRA AGKCDAVFKGFSDCLLKLGDSMANYPQGLDDKT
                    *****.**** *:***::*:*:***:* :*:*****:*:***:* ***.:*****.*::* 

Xenopus             NLDTICSYWDDFHVCTVTALADCQEGAADIWEKLKRQSKNLNIQGSLFELCPGSA GAPGQ
Gallus              NLQTICAYWDDFHACTLTALTDCQEGATDLWEKLRRESKNLDFQGSLFELCGGGS GAAPS

Oryctolagus         NIKTVCTYWEDFHSCTVTALTDCQEGAKDMWDKLRKESKNLNIQGSLFELCGSGN GAAGP
Mus                 NIKTVCTYWEDFHSCTVTALTDCQEGAKDMWDKLRKESKNLNIQGSLFELCGSSN GAAGS
Homo                NIKTVCTYWEDFHSCTVTALTDCQEGAKDMWDKLRKESKNLNIQGSLFELCGSGN GAAGS
                    *:.*:*:**:*** **:***:****** *:*:**:::****::******** ..  **

Xenopus             RLLFPAFLPLLMVFLSTLFIWVLQ-      144
Gallus              L-LPPALPVLLGALGAALATWGLPF      144

Oryctolagus         L-L-PALPVLLVSLWAALVTWL-PF      142
Mus                 L-L-PALSVLLVSLSAALATWF-SF      142
Homo                L-L-PAFPVLLVSLSAALATWL-SF      142
                      * **:  **  : ::*  *
```

Figure S1. Alignment of neuritin amino acid sequences of diverse species. Shown are the sequences of Xenopus laevis (NM_001085688), chicken (Gallus gallus, NM_001198731), rabbit (Oryctolagus cuniculus, XM_002720948), mouse (Mus musculus, NM_153529), and human (Homo sapiens, AF136631), respectively. Underlined are the sequences of the peptides NrnA (position 47 to 61) and NrnB (position 83 to 100). The markup line uses a space for a mismatch or a gap, '.' for any small positive score, ':' for a similarity which scores more than 1.0, the program used was CLUSTAL 0(1.2.4), https://www.ebi.ac.uk/Tools/msa/clustalo/. The sequence of the mature protein is given by the block in the middle (position 28 to 115), the amino acid residues of the suggested leader peptides (first 27 residues) as well as those that are removed once the GPI-anchor is added can be seen to the left, and to the right, respectively. Please note that with respect to the mature protein the lower three sequences (separated by the small gap from the upper two ones) differ from each other only at positions 113 and/or 114 (bold letters). This also applies to the sequences of rat, cattle, sheep, camel and donkey (not shown).
